# Biogeographic position and body size jointly set lower thermal limits of wandering spiders

**DOI:** 10.1002/ece3.7286

**Published:** 2021-03-05

**Authors:** Jérémy Monsimet, Hervé Colinet, Olivier Devineau, Denis Lafage, Julien Pétillon

**Affiliations:** ^1^ Department of Forestry and Wildlife Management Inland Norway University of Applied Sciences Koppang Norway; ^2^ CNRS ECOBIO [(Ecosystèmes, biodiversité, évolution)] ‐ UMR 6553 University of Rennes Rennes France; ^3^ Department of Environmental and Life Sciences/Biology Karlstad University Karlstad Sweden

**Keywords:** climate change, *Dolomedes*, fishing spiders, freezing, supercooling ability

## Abstract

Most species encounter large variations in abiotic conditions along their distribution range. The physiological responses of most terrestrial ectotherms (such as insects and spiders) to clinal gradients of climate, and in particular gradients of temperature, can be the product of both phenotypic plasticity and local adaptation. This study aimed to determine how the biogeographic position of populations and the body size of individuals set the limits of cold (freezing) resistance of *Dolomedes fimbriatus*. We compared *D. fimbriatus* to its sister species *Dolomedes plantarius* under harsher climatic conditions in their distribution range. Using an ad hoc design, we sampled individuals from four populations of *Dolomedes fimbriatus* originating from contrasting climatic areas (temperate and continental climate) and one population of the sister species *D. plantarius* from continental climate, and compared their supercooling ability as an indicator of cold resistance. Results for *D. fimbriatus* indicated that spiders from northern (continental) populations had higher cold resistance than spiders from southern (temperate) populations. Larger spiders had a lower supercooling ability in northern populations. The red‐listed and rarest *D. plantarius* was slightly less cold tolerant than the more common *D. fimbriatus*, and this might be of importance in a context of climate change that could imply colder overwintering habitats in the north due to reduced snow cover protection. The lowest cold resistance might put *D. plantarius* at risk of extinction in the future, and this should be considered in conservation plan.

## Graphical abstract

Climate varies along clinal gradients and influences phenotypic plasticity, local adaptations, and associated physiological responses of most terrestrial ectotherms. We investigated and compared the cold tolerance of fishing spiders from contrasting climate. We found a higher cold resistance in *Dolomedes fimbriatus* from northern populations than southern populations. We also found that the red‐listed *Dolomedes plantarius* was less cold resistant, which might put the species at risk in the future because its overwintering habitats may be colder.

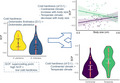

## 1 INTRODUCTION

The ability of a species to cope with variations in abiotic conditions influences its distribution range (Gaston, 2003). Abiotic factors, and among them temperature, shape the geographic range of ectotherm species, and this is even more relevant in the context of global warming (Addo‐Bediako et al., 2000; Somero, 2012). Some ectotherms survive extracellular freezing of their body fluids and are thus freezing tolerant, whereas most ectotherms are freezing intolerant. Instead of having high supercooling abilities (i.e., low supercooling point, SCP), freezing tolerant species, like some alpine species, tend to freeze at relatively high subzero temperatures, a phenomenon that occurs thanks to the synthesis ice nucleators and cryoprotectants that respectively induce and protect against freezing stress (Bale, 2002; Duman, 2001; Duman et al., 2004). Freezing intolerant arthropods, which include freeze‐avoidant, chill‐tolerant, chill‐susceptible, and opportunistic‐survival classes, can exhibit deep supercooling ability, ranging from −15 to −25°C (Danks, 2004), by producing cryoprotectants (e.g., polyols) and antifreeze proteins (Bale, 2002; Duman, 2001).

Body size influences and is influenced by the animal's stage, its body fat content or the concentration of ice‐nucleating bacteria, which affect the SCP (Colinet et al., 2007; David & Vannier, 1996; Johnston & Lee, 1990). The size of animals also changes along latitudinal and altitudinal clines. Both an increase and a decrease of body size toward northern latitudes were observed and theorized under the Bergmann and converse Bergmann rules, respectively (Blanckenhorn & Demont, 2004). For ectotherms, these two rules were first opposed (Mousseau, 1997; Voorhies, 1996), but it seems that both larger and smaller individuals at northern latitudes are possible and the two rules are eventually not exclusive (Blanckenhorn & Demont, 2004), possibly coexisting in close species (e.g., in artic wolf spiders, see Ameline et al., 2018). The latitudinal size cline is of importance as body size also influences cold hardiness (Ansart et al., 2014), for example, with smaller arthropods having better supercooling capabilities than larger ones (Colinet et al., 2007; David et al., 1996; Sinclair et al., 2009; Sømme, 1982). Hence, a negative relationship between ectotherms size and the ability to supercool has been reported (Lee & Costanzo, 1998). Consequently, smaller individuals could benefit from colder temperatures under harsher winter conditions at northern latitudes.

Latitude, by influencing winter conditions, also influences the cold hardiness strategies defined by Bale (1996). Indeed, it influences the temperature gap between the SCP and the lower lethal temperature (Addo‐Bediako et al., 2000; Vernon & Vannier, 2002), and consequently, opportunistic‐survival animals are mainly found in tropical and semitropical regions, chill‐susceptible and chill‐tolerant in temperate and subpolar regions, and freeze‐avoidant in region with severe cold winter conditions.

Despite the importance of latitude on cold resistance, most studies investigating latitudinal clinal changes of arthropods’ physiological tolerance focused on differences between species rather than among populations of the same species (Spicer & Gaston, 1999 but see, e.g., Jensen et al., 2019). Basal cold tolerance is a physiological trait that has evolved many times in arthropods (Sinclair et al., 2003). Most of the knowledge on cold tolerance of arthropods comes from the study of insects, and different mechanisms might influence the cold hardiness of insects versus arachnids. Indeed, Anthony and Sinclair (2019) showed divergent cryoprotective dehydration, the action of losing water by evaporation at low temperature, between insects and arachnids, and the presence of coma under hypoxic conditions is also remarkable in spiders (Pétillon et al., 2009). The same cold hardiness classes are used to categorize freezing intolerance of spiders and insects. Indeed, some spiders are freeze‐avoidant, others chill‐tolerant or chill‐susceptible (Anthony et al., 2019; Kirchner, 1973). However, not all spiders are freezing tolerant (Nentwig, 2012).

In this study, we focused on fishing spiders (Araneae, Pisauridae) with contrasted distributions. These spiders are represented by two species only in Europe. Both species are quite widespread but climatic and habitat conditions in the future might negatively impact their abilities to cope with climate change (Monsimet et al., 2020), especially for the red‐listed *Dolomedes plantarius*. Estimating the cold resistance is essential to adopt efficient conservation strategies for species having a subnivean winter habitat that might become colder in their northern range, Fennoscandia (Wipf & Rixen, 2010).

The thermal performance of populations could be depicted by a thermal performance curve representing how a temperature gradient influences arthropod activity (Sinclair et al., 2012, 2015). However, the estimation of thermal performances requires many individuals per population. Consequently, measuring an anchor point like the SCP is useful to assess the cold tolerance class of species. The SCP represents the lower lethal temperature (LLT) for freezing‐avoidant species and is still a useful indicator for chill‐tolerant species as SCP and LLT are almost similar for them (Bale, 1996). Even though the ecological value of the SCP has been debated (e.g., Ditrich et al., 2018; Renault et al., 2002), it is a useful metric to explore and describe the cold tolerance strategy of poorly studied species (Sinclair et al., 2015), such as *Dolomedes*.

In this study, we assessed the variation in cold resistance, estimated through SCP ability of different populations of the most common *Dolomedes fimbriatus* with contrasted distributions. Due to the rarity of *D. Plantarius*, we decided to sample this species only in northern population. We hypothesized that (a) northern populations of *Dolomedes fimbriatus* have lower SCP values than southern populations, (b) the size of spider in the north is positively related to the SCP, and (c) *D. fimbriatus* has lower SCP values than *D. plantarius*, potentially impacting the distribution of the latter one, less spread in Europe.

## 2 MATERIALS AND METHODS

### 2.1 Case study species and sampling locations

The fishing spiders, *Dolomedes plantarius* and *Dolomedes fimbriatus,* are widespread in Europe with a northern range limit in Fennoscandia. *D. plantarius* has a lower population density and is red‐listed at the European scale (Baillie et al., 1996). The latitudinal contrast encompassed two different biogeographic positions, characterizing two different climatic areas (continental, coded C hereafter versus temperate, coded T). The continental climate is annually colder, and colder months are much colder than under temperate climate (Table 1). Moreover, the variation of temperature among seasons is much higher for continental climate (Table 1).

**TABLE 1:** Climatic characteristics of the sampling sites

Site	Mean temp (°C)	Diurnal range (°C)	Temp seasonality	Coldest month (°C)	Coldest quarter (°C)
T1	11.62	7.03	457.72	3.30	6.27
T2	11.14	6.30	440.90	3.40	6.07
C1	2.56	9.50	865.37	−12.70	−8.25
C2	5.52	8.54	787.69	−7.90	−4.07
C3	6.05	7.78	741.83	−6.90	−2.67

Note

Mean temp: annual mean temperature; Diurnal range: mean diurnal range (mean monthly (maximum temperature – minimum temperature)); Coldest month: mean temperature of the coldest month; Coldest quarter: mean temperature of the coldest quarter (extracted from Worldclim2; see Fick and Hijmans (2017)).

**FIGURE 1:**
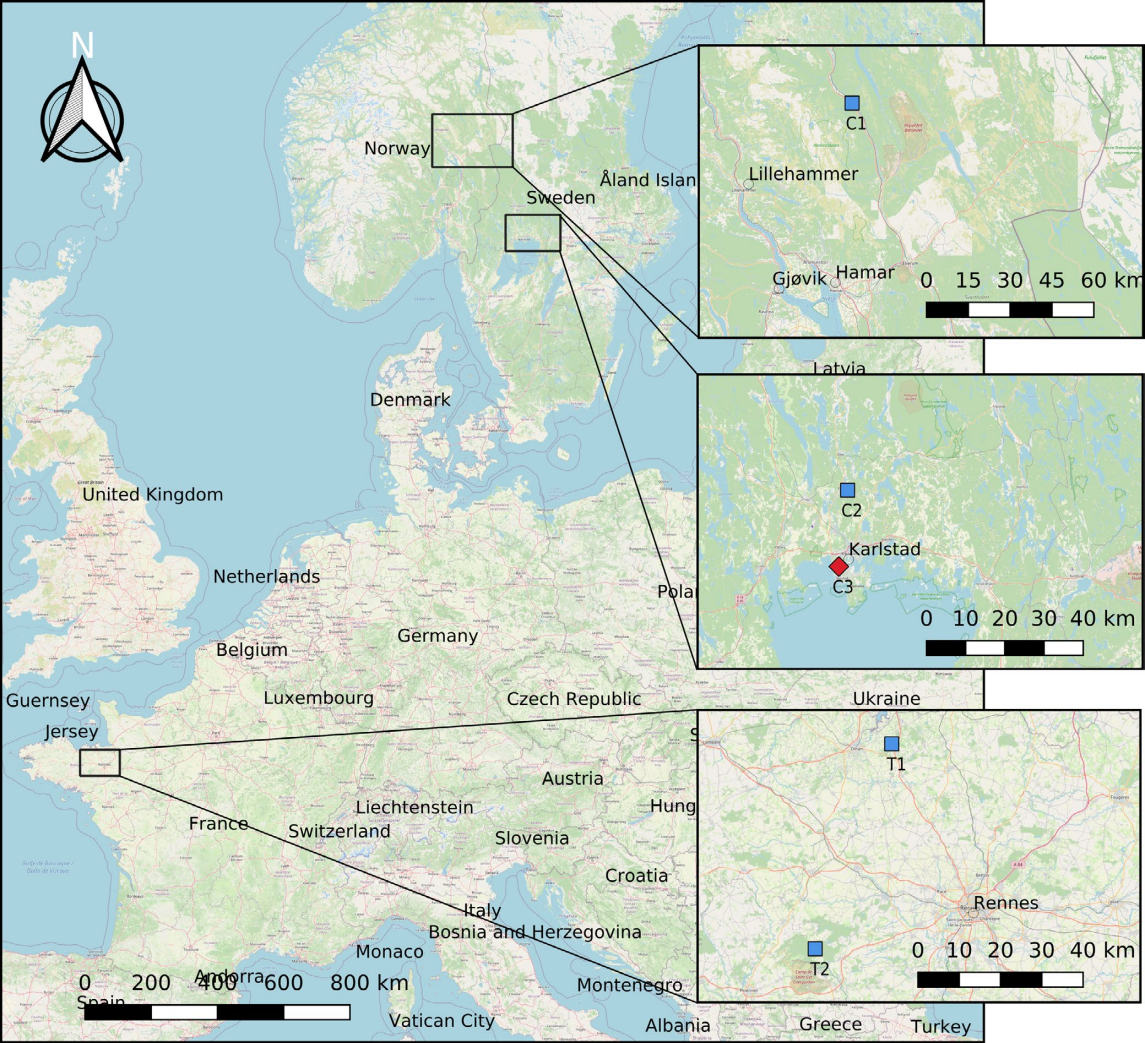
Location of sampling sites for *Dolomedes fimbriatus* (blue squares) and *Dolomedes plantarius* (red square) in France and Fennoscandia

We sampled *D. fimbriatus* individuals of sampled at their range limit and compared them with others from a central latitude of the distribution. We sampled two sites with *D. fimbriatus* in Fennoscandia (C1 and C2; Figure 1), which characterize the northern population, subject to a continental climate. In addition, we sampled two sites in France (T1 and T2; Figure 1), representing the centrally distributed populations exposed to a temperate climate. Given the conservation status of *D. plantarius* in Europe, the limited knowledge on the species, we chose to limit our sampling of this species to the area where it is most abundant (Fennoscandia) and we sampled only one population later compared to its sister species *D. fimbriatus*.

As the SCP is influenced by the developmental stage (Aitchison, 1984; Anthony et al., 2019), we sampled only juvenile spiders of both sexes. The peak of the breeding season of European *Dolomedes* is in late July (Smith, 2000). Females keep egg sacs several weeks before building a nursery web where eggs will hatch and from which spiderlings will later spread out into the surroundings. Juvenile spiders overwinter, but not adults, similarly to other species in the genus (Guarisco, 2010). We sampled *D. fimbriatus* by sweep‐netting the vegetation on sunny and windless days. We sampled *D. plantarius* on the water surface by visual hunting, and active hunting by perturbing the water surface. We sampled, and latter tested the SCP of about 24 spiders at each sampling site (*N* = 24, 24, 21, 26, 24 for C1, C2, C3, T1, T2, respectively, Table 2).

**TABLE 2:** Description of the climatic conditions at the sampling sites, based on the Köppen–Geiger climate classification (Kottek et al., 2006)

Sites	Species	*N*	Country	Climate	SCP (°C)	Body size (mm)
C1	*D. fimbriatus*	24	Norway	Continental	−9.08 ± 0.45	4.13 ± 0.52
C2	*D. fimbriatus*	24	Sweden	Continental	−9.06 ± 0.4	4.43 ± 0.56
C3	*D. plantarius*	21	Sweden	Continental	−7.56 ± 0.32	5.36 ± 0.69
T1	*D. fimbriatus*	26	France	Temperate	−7.78 ± 0.4	4.62 ± 0.46
T2	*D. fimbriatus*	24	France	Temperate	−5.39 ± 0.4	4.44 ± 0.48

Note

*N*: number of spiders tested; SCP: mean SCP ± *SD*; Body size: mean length of the carapace ± *SD*.

### 2.2 Measurement of the supercooling point

**FIGURE 2:**
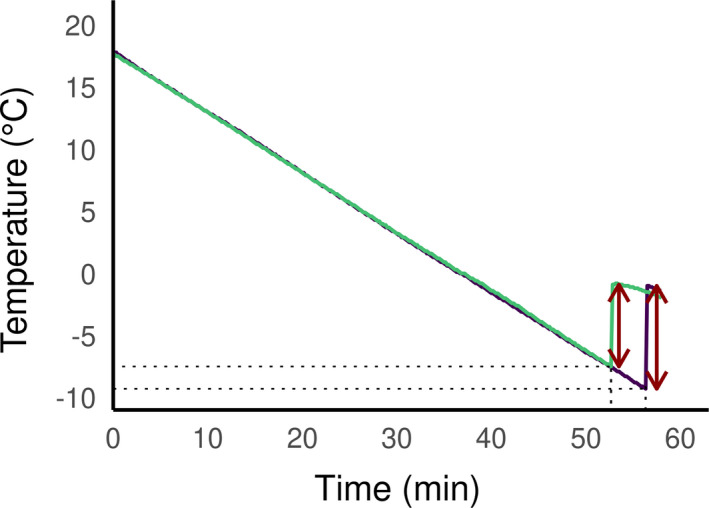
Cooling curves of *D. plantarius* (one spider from C3, in yellow) and *D. fimbriatus* (one spider from C2, in purple) recorded during a cooling experiment. The SCP (dotted line) is followed by the exotherm (dark red arrows), a sudden increase in the measured temperature due to the release of latent heat linked to the phase change during freezing

To determine the SCP, we placed the spiders in centrifuge tubes, which were submerged in a cryostat bath (Polystat CC3, Huber Kältemaschinenbau AG, Germany) filled with heat transfer fluid (Thermofluid SilOil, Huber, Germany). The temperature of the bath was slowly reduced at a rate of 0.5°C/min to reach a target temperature of −30°C. To monitor the temperature of the spiders, we placed a K‐type thermocouple in direct contact with the spider opisthosoma, secured with Parafilm® and connected to a Testo 175T3 temperature data logger (Testo SE& Co., Germany). We recorded the temperature every ten seconds. The SCP was defined as the temperature at the onset of the freezing exotherm produced by the latent heat (see Figure 2 for representative exotherms), and we considered spiders dead if they did not move in the 24 hr after SCP test.

The number of spiders tested per day was limited by the capacity of the instrument (4 spiders at a time). Therefore, we included the time lag between capture and tested it to account for possible acclimation to laboratory conditions in our models (variable Diff).

### 2.3 Measurement of spider body size

We measured the spiders’ body size after the SCP experiment to avoid injuring the spiders and biasing the results. We took a picture of the spider’ back together with a measuring tape for measuring the body size later in the ImageJ software (Schneider et al., 2012). We measured the highest length and largest width of the carapace (prosoma) which are commonly used as proxy for whole body size, fitness, and metabolic rate in spiders (Jakob et al., 1996; Penell et al., 2018).

### 2.4 Data treatment

The carapace width and length were highly correlated (*γ* = 0.83, Pearson correlation test), so we used the carapace length as a proxy of body size (Jakob et al., 1996) and referred to as body size hereafter. Moreover, all the climatic variables presented in Table 1 were highly inter correlated and correlated to the latitude (*γ* > 0.9), so we kept the latitude and categorical climate as a proxy of climatic variables.

We modeled the SCP of the four *D. fimbriatus* (model modClim hereafter) with several candidate linear models including predictor variables Diff (time between capture and SCP measurements), site, climate (continental/temperate, as defined by the biogeographic location), sex, and body size. We also considered the interaction between climate and body size and/or the interaction between body size and site (See Appendix S1 for the list of candidate models). We modeled the SCP of species from Scandinavia (*D. fimbriatus* and *D. Plantarius*, model modSp in the following) to compare the SCP of species from northern populations. We modeled the SCP with several candidate linear models with variables Diff, site, species, sex, and body size, as well as the interaction between species and body size and/or the interaction between body size and site (See Appendix S2 for the list of candidate models).

### 2.5 Statistical analysis

We used packages rstanarm (Goodrich et al., 2020), modelbased (Makowski et al., 2020), and bayestestR (Makowski et al., 2019) in R (R Core Team, 2020) to fit the linear models in a Bayesian framework. We used a normal distribution centered on 0 and a standard deviation of 2.5 as weakly informative priors (rather than using flat priors, see Gelman et al., 2008; Gelman & Shalizi, 2013). We fitted the models using four chains and 4,000 iterations. We used leave‐one‐out cross‐validation value (LOO value) to compare the predictive accuracy of fitted models, and to select the most accurate model (Vehtari et al., 2017). We checked the convergence of the models both visually and by making sure that Rhat value was not larger than 1.01 (Vehtari et al., 2020).

Following Makowski, Ben‐Shachar, and Lüdecke (2019 and 2019), we represented the median of the posterior distribution and its uncertainty with a credible interval of 95%. We used both the probability of direction (pd), which is the probability that the posterior distribution of a parameter is strictly positive or negative, and the percentage of the full region of practical equivalence (ROPE). The thresholds beyond which the effect was considered as significant (i.e., non‐negligible) were pd > 95% and ROPE < 2.5%.

## 3 RESULTS

### 3.1 General results

The SCP of the spiders varied from −2.6 to −16.4°C, with an average of −7.8 ± 2.3°C (*N* = 119). Figure 2 shows typical cooling curves of *Dolomedes fimbriatus* (from C2) and *Dolomedes plantarius* (from C3) with exotherms of about 8 and 6.5°C and a SCP of −9.3 and −7.5°C, respectively. None of the spiders tested survived freezing.

The body size of the sampled juveniles of *D. fimbriatus* was 4.28 ± 0.56 mm in the south and 4.53 ± 0.47 mm in the north and did not significantly differ between sites (ROPE > 2.5%). The body size of juveniles of *D. plantarius* was on average 5.36 ± 0.69 mm and did not significantly differ from *D. fimbriatus* juveniles (pd > 95% but ROPE > 2.5%).

### 3.2 Validation and selection of models

All of our candidate models converged (Rhat < 1.01). According to LOO values, some models were considered equivalent (Appendices S1 and S2). The modClim model with the lowest LOO value and therefore the highest predictive power included variables Diff (time between capture and test), climate, body size, and the interactive effect of climate and body size (Table 3). For modSp model, the best model included Diff, species, body size, and the interactive effect of body size and species.

**TABLE 3:** Parameter estimates of the most accurate model explaining the SCP values between different climatic areas for *D. fimbriatus* (modClim, see Appendix S1)

	Estimate	CI low	CI high	pd	ROPE (%)	Rhat
(Intercept)	−8.10	−12.40	−3.92	1.00	0.01	1.00
Diff	−0.26	−0.40	−0.12	1.00	35.80	1.00
Temperate	8.58	2.44	14.51	1.00	0.10	1.00
Body size	6.88	−2.20	16.05	0.93	1.26	1.00
Temperate:Body size	−15.37	−28.90	−1.82	0.99	0.11	1.00

Note

CI, 95% credible intervals; Diff, time difference between date of capture and date of test; pd, probability of direction; ROPE, percentage of the full region of practical equivalence; Temperate, climate variable (continental climate in the intercept); Temperate:Body size, interactive effect of the climate and body size.

### 3.3 Comparison of SCP across latitudes and between species

**FIGURE 3:**
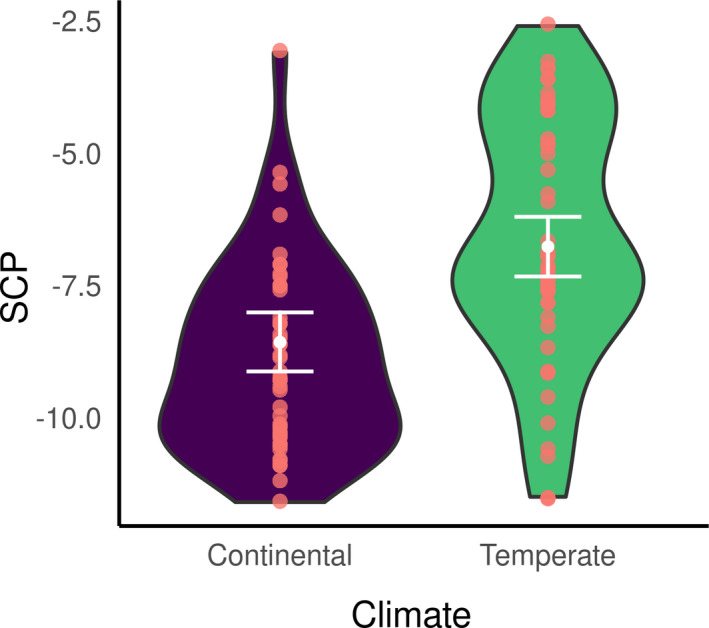
Marginal posterior means of SCP (white dot) estimated under modClim for the two different climatic areas and its 95% credible interval (white bar). Red dots represent the original data, and the violin distributions represent a density plot

**FIGURE 4:**
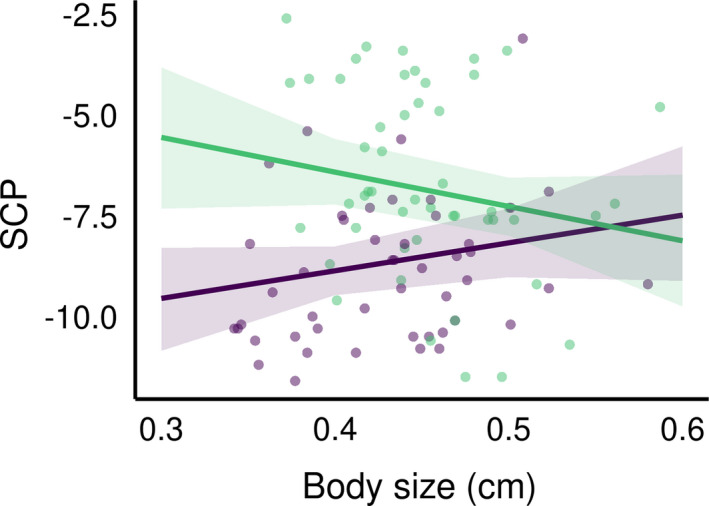
Predicted effect of *D. fimbriatus* body size on the SCP, and its 95% credible interval, for the two different climatic areas under modClim. Purple: predictions for the continental climate, green: predictions for the temperate climate; dots represent original data

Regarding *D. fimbriatus* (modClim; Table 3), the SCP of individuals of southern and northern populations significantly differed (pd > 95%, <2.5% in ROPE, Figure 3) and was −6.6 ± 2.3°C (min. −11.5°C, max. −2.6°C; *n* = 50) and −9.05 ± 2.31°C (min. −6.30°C, max. −2.30°C, *n* = 48), respectively. The effect of spiders' body size on the SCP was significantly different between the two climatic areas (pd > 95%, <2.5% in ROPE; Figure 4). Namely, the SCP increased with the body size of spiders in the northern climate (median = 6.88 [−2.18; 16.05]) while the SCP decreased with the body size in the South (median = −8.52 [−19.06; 2.40]), which means that larger spiders had, in the northern and southern climate, respectively higher and lower SCP than smaller spiders.

**FIGURE 5:**
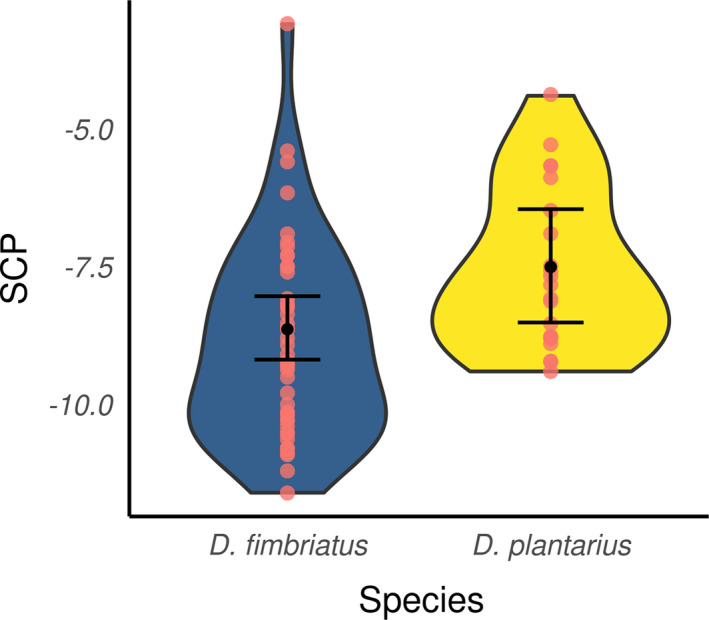
Marginal posterior means of SCP (black dot) estimated under modSp for the two different species and its 95% credible interval (black bar). Red dots represent the original data, and the violin distributions represent a density plot

Regarding ModSp (Table 4), the SCPs of individuals of *D. plantarius* and *D. fimbriatus* of northern populations significantly differed (pd > 95%, <2.5% in ROPE, Figure 5) and were higher for *D. plantarius* (−7.56 ± 0.32 min. −9.4°C, max. −4.4°C; *n* = 21; for *D. fimbriatus*, see above). Nonetheless, the effect of body size on the SCP was not significant (pd < 95%) and we did not find a significant effect of Diff for modSp (pd < 95%).

**TABLE 4:** Parameter estimates of the most accurate model explaining the SCP values between the two species in continental climate (modSp, see Appendix S2)

	Estimate	CI low	CI high	pd	ROPE (%)	Rhat
(Intercept)	−10.19	−14.50	−5.90	1.00	0.00	1.00
Diff	−0.11	−0.32	0.10	0.83	74.44	1.00
*D. plantarius*	5.10	−1.14	10.90	0.95	1.15	1.00
Body size	6.57	−1.84	14.10	0.94	1.25	1.00
*D. plantarius*:Body size	−8.62	−20.33	3.80	0.92	0.76	1.00

Note

CI, 95% credible intervals; Diff, time difference between date of capture and date of test; *D. plantarius*, species variable (*D. fimbriatus* in the intercept); *D. plantarius*:Body size, interactive effect of species and body size; pd, probability of direction; ROPE, percentage of the full region of practical equivalence.

## 4 DISCUSSION

Our study showed that the SCP of northern *D. fimbriatus* from a continental climate was lower than the SCP of southern populations from a temperate climate. The SCP was positively related to body size in the north, and the opposite effect was observed in southern population of *D. fimbriatus*. Finally, we found that the SCP of *D. fimbriatus* was lower than that of *D. plantarius*, even though the juveniles tested did not differ in size.

The SCP of *D. fimbriatus* decreased with increasing latitude, while juveniles of the species did not differ in size. In this study, we tested four populations from two biogeographic locations that were characterized by different climates and latitudes along the species distribution range. The northern populations, at the range limit, experience cold winters with permanent snow cover, whereas the southern populations of *D. fimbriatus*, from a more central latitude of the range, experience warmer winters with only rarely a snow cover. The northern and southern locations are characterized by temperate and continental climate, respectively (Kottek et al., 2006) and the corresponding range of temperatures might explain the decrease in SCP toward the North. Indeed, temperature influences cold hardiness in arthropods, including spiders (Nentwig, 2012) and a poleward increase in thermal tolerance is observed in many ectotherms (Sunday et al., 2011). An acclimation to warmer temperatures, as for southern spiders, can also reduce the tolerance to cold conditions (Jensen et al., 2019). At the same time, northern *D. fimbriatus* could benefit from their cold acclimation by being more active during cooler periods in summer (Everatt et al., 2013). Indeed, according to the metabolic cold adaptation (MCA) hypothesis, individuals from higher latitude have higher metabolic rate at a given temperature (Clarke, 1991, 1993) by showing clinal latitudinal variation in enzymes associated with cold tolerance (Sinclair, 2002).

The impact of diurnal activity range, together with temperature, is essential cues to determine the cold resistance of ectotherm arthropods (e.g., soil dwelling collembolan *Orchesella cincta* see Jensen et al., 2019, or Paaijmans et al., 2013, Seebacher et al., 2015). These might have impacted spiders differently at the time of our experiments (late summer/ early autumn), as northern *D. fimbriatus* are confronted to earlier and harsher winter. Indeed, the supercooling varies at the individual scale during the season, mainly due to the variation in concentration of cryoprotectants during the year (Sømme, 1982). These two cues have been shown to impact the overwintering of another *Dolomedes* species, from North America (*D. triton*; Spence & Zimmermann, 1998), and might similarly impact the overwintering of *D. fimbriatus*. To our knowledge, *Dolomedes* species are inactive during winter (Aitchison, 1984). Schmidt (1957) noted that *D. fimbriatus* overwinters twice before reaching the adult stage. He also noted that juveniles spend the winter in dry vegetation at high strata, which is probably the overwintering habitat of the southern spiders we tested here. However, the northern *Dolomedes* we tested endure temperatures colder than the SCP measured in this study. For this reason, we hypothesized that, similarly to *Dolomedes triton* in Canada (Spence & Zimmermann, 1998), spiderlings and juveniles overwinter under the snow. Indeed, the temperature in the subnivean layer, which is between the soil surface and the base of the snowpack, is warmer and more stable than the air temperature above the snow, and protect species from temperatures lower than their SCP (Marchand, 1982).

*Dolomedes*, like other spider species, are not freezing tolerant as none of the spiders tested survived freezing. Cold hardiness of *Dolomedes* is important for winter survival. Based on the cold hardiness classification of Bale (1996, 2002) (see also Appendix S3 for a summarized classification), we hypothesize that both *Dolomedes*, at least from the northern populations, could be either chill‐susceptible or freeze‐avoidant. The main difference between these two cold hardiness classes is the ability to survive damages caused by cold injuries. Freezing‐avoidant species survive until freezing point, while chill‐tolerant die at moderately low but not freezing temperatures due to chill injuries. The proximity of the LLT or CTmin (critical thermal minimum) and SCP detected in spiders from a close family (*Pardosa*, Lycosidae) at northern latitudes (Anthony et al., 2019) let us predict that *Dolomedes* are most probably chill‐susceptible. Nonetheless, we only tested the SCP and more measurements, such as the lower lethal temperature, would be necessary to define the cold hardiness class more precisely. The cold hardiness class of *Dolomedes* might also vary between the two biogeographic positions as demonstrated for the butterfly *Piries rapae* which is either freeze‐tolerant or freezing‐avoidant depending on the latitude (Li & Zachariassen, 2007).

Even if *D. fimbriatus* from the two bioclimatic areas did not differ in body size, we found an increase of the SCP with increasing spider body size for the northern populations. Smaller individuals being more cold tolerant than bigger ones is a general trend for ectotherm animals (e.g., for ants see Hahn et al. (2008), for beetles see Johnston and Lee (1990)). This trend is also observed for spiders with smaller instars being more cold tolerant than larger juveniles and adults (Almquist, 1970; Bayram & Luff, 1993).

The converse effect was observed in southern *D. fimbriatus* with a decrease in SCP with increasing body size. This difference in strategy between temperate and colder habitats has been reported in other species from the closely related family of Lycosidae (Ameline et al., 2017). The northern spiders have a shortened breeding season, which can impact life‐history traits such as body size (Bowden et al., 2015). The smaller *D. fimbriatus* under continental climate could be advantaged as they can survive colder winters. After the winter, northern fishing spiders could accelerate their development because cold‐adapted ectotherms have a higher metabolic rate in an environment with limited energy (Sinclair et al., 2012). Moreover, this pattern might illustrate the clinal variation in life duration. Some *Dolomedes spp*. live one year (see Bonnet, 1930), while others from northern latitudes two years or more (see Duffey, 2012; Spence & Zimmermann, 1998). The northern *D. fimbriatus* would overwinter at a smaller size than southern individuals. The spiders from temperate climate have a longer time window to grow, and they were still growing when we sampled them and this might have resulted in smaller adult body size. Indeed, European spiders from northern latitudes tend to be smaller than spiders from lower latitudes (Hein et al., 2019; Puzin et al., 2014). The absence of difference in body size between latitudes here might be due to a limit in our sampling method, indirectly targeting individuals with similar body size. This might hide an effect of the age (impact of the instar) of spiders on the SCP.

The SCPs measured in this study were close to those measured for phylogenetically close spiders (from the same Lycosoidea superfamily) from northern latitudes (Anthony et al., 2019). These values are considered as medium cold resistance (Nentwig, 2012). Nonetheless, we found slightly higher resistance to cold temperature in *D. fimbriatus* compared to *D. plantarius* (for populations from similar biogeographic areas). Although it seems that *D. fimbriatus* have smaller size, we could not detect a significant difference. Nonetheless, this tendency would confirm the observation that specialist species are larger under harsher conditions because they are more adapted to their environment (Ameline et al., 2018). In turn, the difference between species might explain the wider northward distribution of *D. fimbriatus* compared to that of *D. plantarius*. The former could have benefited from higher cold resistance to expand and increase survival of populations in coldest areas, and this might explain the more limited distribution range of *D. plantarius*, abilities to tolerate cold being an important factor to explain past colonization (Sunday et al., 2011). Nonetheless, a better knowledge of the phylogeny of European fishing spider that is unfortunately still poorly documented (see Macías‐Hernández et al., 2020; Piacentini & Ramírez, 2019; Tanikawa & Miyashita, 2008) would allow us to conclude on the difference in species adaptation to climate by using a phylogenetic comparative method (Blomberg & Garland, 2002; Garland & Adolph, 1994).

Climate change impacts spiders in various ways. At northern latitudes, subnivean layer is supposedly a nonfreezing environment with quite stable temperatures (Pruitt, 1957) but snow density and length of the snow season impacts the stability of these conditions (Bale & Hayward, 2010; Pauli et al., 2013). While air temperature increases with climate change, the subnivean layer may become colder (Wipf & Rixen, 2010). This paradox is already negatively affecting invertebrates (Slatyer et al., 2017; Williams et al., 2015). Even though we found that fishing spiders from continental climate tolerated colder temperatures than spiders from temperate climate, the lowest SCP was higher than the lowest air temperature measured historically in Fennoscandia. A weakened subnivean shelter could negatively influence northern populations and even more so for the rare *D. plantarius* which is less cold resistant. Another impact of the increased length of the snow free season could be a second clutch in northern *Dolomedes*, as reported in the arctic Lycosidae *Pardosa glacialis* (Høye et al., 2020).

We found that the cold tolerance of fishing spiders varied among populations, between climates for *Dolomedes fimbriatus*, northern spiders being acclimated to colder climate. Moreover, we found lower SCP for *D. plantarius*, which might be important to consider for the conservation of this red‐listed species (Baillie et al., 1996). Indeed, the impact of a smaller snow layer might negatively impact the future distribution of both species in the northern part of their distribution. Moreover, the distribution of understudied invertebrates can be explored and predicted by studying life‐history traits like cold resistance (Mammola et al., 2020), especially by explicitly integrating the ecophysiology of species into distribution modeling.

## CONFLICT OF INTEREST

The authors declare no conflict of interest.

## AUTHOR CONTRIBUTION

**Jérémy Monsimet:** Conceptualization (equal); Formal analysis (lead); Investigation (equal); Methodology (equal); Writing‐original draft (lead); Writing‐review & editing (equal). **Hervé Colinet:** Conceptualization (equal); Methodology (lead); Resources (equal); Writing‐review & editing (equal). **Olivier Devineau:** Conceptualization (supporting); Formal analysis (equal); Writing‐review & editing (equal). **Denis Lafage:** Conceptualization (supporting); Investigation (equal); Writing‐review & editing (equal). **Julien Pétillon:** Conceptualization (equal); Investigation (equal); Methodology (lead); Resources (equal); Writing‐review & editing (equal).

## Supporting information

Appendix S1‐S3

## Data Availability

All the data and R scripts to analyze them are available on Dataverse (https://doi.org/10.18710/N3P4TH).
