# Supplementary material for: Biogeographic position and body size jointly set lower thermal limits of wandering spiders
Source: Ecol Evol. 2021 Mar 5;11(7):3347–56. doi: 10.1002/ece3.7286 (PMC8019051; doi:10.1002/ece3.7286)
Supplement: Supplementary file 1 — Appendix S1‐S3 [file ECE3-11-3347-s001.docx]

# Appendices

Appendix 1: Comparison of the expected predictive accuracy of models comparing the two geographic areas (modClim). Difference of expected log predictive density and standard error between model for the leave-one-out cross validation (LOO) are shown (epld: expected log predictive density, SE: Standard-error of the epld).

| Code | Model | elpd_diff | se_diff |
| --- | --- | --- | --- |
| mC11 | SCP ~ Diff + Climate + Body size + Climate:Body size | 0.00 | 0.00 |
| mC2 | SCP ~ Diff + Site | -0.01 | 2.75 |
| mC4 | SCP ~ Diff + Site + Climate | -0.13 | 2.75 |
| mC8 | SCP ~ Diff + Climate + Sex + Body size + Climate:Body size | -0.33 | 1.13 |
| mC5 | SCP ~ Diff + Site + Climate + Sex | -0.48 | 3.00 |
| mC12 | SCP ~ Diff + Site + Body size + Site:Body size | -0.70 | 1.86 |
| mC10 | SCP ~ Diff + Site + Climate + Sex + Body size + Climate:Body size + Site:Body size | -0.94 | 2.22 |
| mC9 | SCP ~ Diff + Site + Sex + Body size + Site:Body size | -0.97 | 2.29 |
| mC3 | SCP ~ Diff + Climate | -1.01 | 2.03 |
| mC6 | SCP ~ Diff + Site + Climate + Body size | -1.27 | 2.82 |
| mC7 | SCP ~ Diff + Site + Climate + Sex + Body size | -1.63 | 3.07 |
| mC1 | SCP ~ 1+Diff | -18.66 | 6.21 |

Appendix 2: Comparison of the expected predictive accuracy of models comparing the two species (modSp). Difference of expected log predictive density and standard error between models for the leave-one-out cross validation (LOO) are shown (ELPD: expected log predictive density, SE: Standard-error of the ELPD).

| Code | Model | elpd_diff | se_diff |
| --- | --- | --- | --- |
| mS11 | SCP ~ Diff + Species + Body size + Species:Body size | 0.00 | 0.00 |
| mS3 | SCP ~ Diff + Species | -0.11 | 1.72 |
| mS8 | SCP ~ Diff + Species + Sex + Body size + Species:Body size | -0.39 | 1.26 |
| mS12 | SCP ~ Diff + Site + Body size + Site:Body size | -0.99 | 0.71 |
| mS2 | SCP ~ Diff + Site | -1.08 | 1.78 |
| mS10 | SCP ~ Diff + Site + Species + Sex + Body size + Species:Body size + Site:Body size | -1.14 | 1.48 |
| mS4 | SCP ~ Diff + Site + Species | -1.18 | 1.82 |
| mS9 | SCP ~ Diff + Site + Sex + Body size + Site:Body size | -1.30 | 1.35 |
| mS5 | SCP ~ Diff + Site + Species + Sex | -1.34 | 2.11 |
| mS6 | SCP ~ Diff + Site + Species + Body size | -1.99 | 1.47 |
| mS7 | SCP ~ Diff + Site + Species + Sex + Body size | -2.16 | 1.72 |
| mS1 | SCP ~ 1+Diff | -3.01 | 3.27 |

Appendix 3: Cold hardiness classes of exotherm arthropods. LLT: lower lethal temperature, SCP = supercooling point (Bale 1996 and 2002).

| Class | Subclass | Definition |
| --- | --- | --- |
| Freezing tolerant |  | LLT < SCP |
| Freezing avoidant | Freeze-avoidant | LLT = SCP |
|  | Chill-tolerant | LLT ≥ SCP |
|  | Chill-susceptible | LLT > SCP |
|  | Opportunistic-survival | LLT ≫ SCP |
